# Utility of a Simulation-Based Mastery Learning Curriculum for Teaching Labor and Delivery Procedures

**DOI:** 10.7759/cureus.95598

**Published:** 2025-10-28

**Authors:** Gabrielle K Smith, Shivani Arza, Christopher S Ennen

**Affiliations:** 1 Department of Obstetrics and Gynecology, University of Virginia, Charlottesville, USA

**Keywords:** competency, mastery learning, medical education, obstetrics, procedural skills, simulation

## Abstract

Introduction: Mastering obstetric procedural skills is difficult. These skills require a tactile understanding of pelvic anatomy, must be performed in real time, and learners’ performance cannot be visualized by coaches, making it difficult to obtain feedback. Simulation-based mastery learning (SBML), a competency-based approach in which learners acquire skills to predefined standards in an environment free of risks, is a potential solution to this dilemma. By incorporating SBML into obstetric procedural skills training, learners have the opportunity to safely develop highly competent skills before engaging in patient care.

Methods: This was a prospective cohort study evaluating the utility of the SBML (DELIVER SBML curriculum) in developing knowledge and comfort with the independent performance of obstetric procedural skills in graduate medical education learners.

Results: All learners (n=11) completed the DELIVER SBML curriculum and were able to successfully perform membrane rupture, cervical exams, fetal scalp electrode (FSE) placement, and intrauterine pressure catheter (IUPC) placement. They underwent a written knowledge assessment of obstetric procedural skills. Pre-SBML curriculum, the median number of questions correct was 9/10 (90%) (interquartile range (IQR) 6 (60%), 10 (100%)). Post-SBML, the median number of questions correct was: 9/10 (90%), (IQR 8 (80%), 10 (100%)), and Wilcoxon signed-rank testing demonstrated no statistically significant difference (p=0.102). Using a 5-point Likert Scale, participants expressed their degree of agreement with the statement, “I feel comfortable independently performing obstetric procedural skills.” Pre-SBML, median ranking was 1/5 (Strongly Disagree), (IQR 1, 2). Post-SBML, median was 4/5 (Agree) (IQR 4,5), with a statistically significant difference noted (p=0.014).

Discussion: Learners have varying degrees of knowledge and comfort regarding obstetric procedural skills. Following completion of the DELIVER SBML curriculum, learners had significantly improved perceived readiness for independence in performing obstetric procedural skills, although no significant differences were noted on scores of written knowledge assessments.

## Introduction

Within obstetrics, procedural skills are necessary for effective labor management. Procedures such as cervical exams, artificial rupture of membranes (AROM), and fetal scalp electrode (FSE) placement must be performed efficiently and accurately to identify and manage labor. These same skills are also critical for management of obstetric emergencies, including umbilical cord prolapse or fetal heart rate tracings consistent with acute fetal compromise [1]. However, obstetric procedural skills are difficult for learners to master. When performed without the presence of an epidural, exams are uncomfortable for patients, limiting the time learners have to identify anatomic landmarks and develop skills [2,3]. Providers similarly have a goal of limiting the number of vaginal procedures and exams performed on a single patient to decrease the risk of infection [4]. Given these challenges, graduate medical education learners frequently require months of training before consistently performing basic obstetric procedures accurately and to an appropriate level of competence. Simulation-based mastery learning, (SBML), is proposed as a solution. SBML is an evidence-based educational concept that improves competency among medical trainees in procedure performance by requiring learners to meet fixed competency standards during skill development, regardless of the time necessary to achieve skills [5]. SBML has previously been used to teach learners to perform cervical cerclage with significant improvement in skill performance [6].

The use of simulation in obstetrics has increased over time and is a mainstay of education at all levels, from undergraduate to graduate medical education (UME and GME, respectively) [6-10]. Simulation has contributed to improved clinical outcomes in many areas, including shoulder dystocia management, forceps-assisted deliveries, and postpartum hemorrhage [8,11-12]. However, for GME learners who also serve as providers and must perform medical procedures correctly and appropriately to ensure patient safety in clinical practice, simulation alone is not sufficient. Thus, incorporating simulation into a mastery learning framework, SBML, is necessary not only to provide learners with a safe space to practice skills but also to give them the opportunity to develop predetermined levels of competence to effectively use their skills in a real-world environment. Individual learner differences impact the teaching process, and these differences are addressed in mastery learning, allowing all learners to achieve set competency standards and a higher level of performance [13]. In deliberate practice, strong, consistent educational interventions are used to improve skills, and this is akin to the simulation-based mastery learning approach demonstrated in this project. Ultimately, mastery learning and deliberate practice can lead to improved health outcomes [14].

Our primary objective in this study was to develop an SBML curriculum to improve GME learners’ comfort, knowledge, and ability to independently perform obstetric procedures on labor and delivery (L&D). By using simulation, we provided a risk-free environment for learners to practice invasive, uncomfortable, and tactile skills. Furthermore, by using a mastery learning framework, we aimed to develop a resource focused on the needs of GME learners who require efficient development of high-level skill competence to ensure patient safety. Of note, this article was previously presented as an abstract and oral presentation at the University of Virginia Innovation in Education Week on March 3rd, 2025, in Charlottesville, Virginia.

## Materials and methods

We implemented the DELIVER SBML curriculum for first-year Obstetrics and Gynaecology (OBGYN), Family Medicine, and Emergency Medicine residents as an opt-in training program at our institution (Table 1). We chose the DELIVER acronym to highlight the skills included in the SBML curriculum, as each skill plays a role in moving a patient through the induction process to a successful vaginal delivery. The curriculum was not a formal part of residency training but rather an optional research study session to which learners were invited to participate. Funding was awarded through the Peyton T. Taylor Grant through the OBGYN Department of our institution. Informed consent for treatment and open access publication was obtained or waived by all participants in this study. Our Institutional Review Board for the Social and Behavioral Sciences approved the study, study number 5728. Pre- and post-course qualitative survey data, and quantitative knowledge-based data were collected to assess learner knowledge, comfort, and confidence with obstetric procedural skills.

**Table 1:** DELIVER acronym expansion

D	Dilation	Assessment of cervical dilation via digital exam
E	Effacement	Evaluation of cervical effacement and consistency
L	Location of Presenting Part	Palpation of fetal station and position during cervical exam
I	Intrauterine Pressure Catheter	Placement of intrauterine pressure catheter
V	Vaginal Balloon Placement (Cervical Foley)	Simulated cervical ripening with transcervical catheter
E	Fetal Scalp Electrode	Placement of fetal scalp electrode
R	Rupture of Membranes	Artificial rupture of membranes using an amniotomy hook

We offered the curriculum via email recruitment to all first-year residents rotating on L&D, totaling approximately 18 learners annually. When approached via email to participate, 11 opted into the program. Learners required no prerequisites - knowledge, qualifications, or training - to prepare for this activity, as we specifically focused training efforts on learners with minimal exposure to obstetric procedures. Facilitators, two upper-level OBGYN residents, were competent in knowledge and performance of the procedures taught in the curriculum, as well as in their ability to coach learners through completion of the curriculum according to a mastery learning model. Facilitators were educated about mastery-based learning techniques, and all facilitators reviewed how to give appropriate feedback and teach using a mastery learning context prior to facilitating the curriculum for learners. They additionally had familiarity with the setup and use of the PROMPT Flex Birthing Simulator and Cervical Dilation and Effacement Module (PROMPT Flex Birthing Simulator and Cervical Dilation and Effacement Module, Limbs & Things, Savannah, GA, Product 80200, 80202).

This course was developed by a senior OBGYN resident with an interest in medical education (GS) and a maternal-fetal medicine physician with a particular interest in medical education and simulation with prior experience and training in these fields (CE). All educational materials described below were created, reviewed, and edited by members of this team. The curriculum included the following: (1) Pre-SBML Course Survey (Appendix 1), (2) standardized lecture reviewing the skills involved in the curriculum (Video 1), (3) simulation-based mastery learning component with skill achievement noted using the Procedural Skills Checklist (Table 2), (4) Post-SBML Course Survey (Appendix 2). The surveys and standardized lectures were reviewed by GS and CE, who have experience in simulation research and assessment, but a formal validation process was not performed. Prior to the simulation session, the PROMPT Flex Birthing Simulator was assembled, and the simulation was trialed with our course development team to determine an estimate of the time necessary for learners to complete the simulation SBML curriculum, as well as the time needed to set up the simulation itself. The SBML curriculum was conducted during learners’ shifts on service in a quiet work area, typically an empty classroom.

**Video 1:** Simulation-based mastery learning lecture Lecture that describes the procedures that will be covered in the mastery learning course.

**Table 2:** Procedural skills checklist Checklist of procedural skills and knowledge required of learners at each step in the DELIVER simulation-based mastery learning (SMBL).

Step in SBML Curriculum	Exam/Skill	Required Competency
Step 1.	Cervical Exam	States the correct cervical dilation: 1 cm
		States the correct cervical effacement: 40%
		States the correct fetal station: -2
Step 2.	Cervical Foley Placement	State ruptured membranes as contraindication (when prompted)
		Place balloon above internal os
		Inflate balloon with 30cc of fluid
Step 3.	Cervical Exam	States the correct cervical dilation: 4 cm
		States the correct cervical effacement: 70%
		States the correct fetal station: -1
Step 4.	Artificial Rupture of Membranes (AROM)	States lack of fetal engagement as contraindication when prompted
		Technically successful amniotomy using amniotic fluid perforator instrument with fluid release
Step 5.	Cervical Exam	States the correct cervical dilation: 6 cm
		States the correct cervical effacement: 100%
		States the correct fetal station: 0
Step 6.	Intrauterine Pressure Catheter (IUPC) Placement	Places catheter on side of uterine wall opposite of placenta
		Catheter visibly hubbed on model between vertex and uterine wall confirming proper placement
Step 7.	Fetal Scalp Electrode (FSE) Placement	FSE lead firmly in place
		FSE not placed on fontanelle
		Able to manipulate clip to release FSE sheath
Step 8.	Cervical Exam	States the correct cervical dilation: 10 cm
		States the correct cervical effacement: 100%
		States the correct fetal station: +1

The Pre-SBML Course Survey (Appendix 1) was administered to assess baseline knowledge, comfort, and understanding of obstetric procedural skills. This included 10 knowledge-based multiple-choice questions focused on how to perform the procedures included in the curriculum, as well as a survey utilizing a 5-point Likert scale to assess comfort with skill performance. The facilitator set up the PROMPT Flex Birthing Simulator with the Cervical Dilatation & Effacement Module in advance for the procedural skills.

Specifically, the following skills were included in our curriculum: digital cervical exam, cervical Foley balloon placement, AROM, and placement of internal monitors (FSE and IUPC). Using the Procedural Skills Checklist above (Table 2), facilitators engaged with learners until they were able to perform each skill proficiently on the simulator, meeting all the pre-determined milestones required for completion of the mastery learning teaching. Each learner completed the series of tasks in sequence, taking approximately 30-45 minutes. 

After the session, learners completed a Post-Course Survey (Appendix 2), including a survey utilizing a 5-point Likert scale to assess post-curriculum comfort, as well as a clinical knowledge assessment using the same 10-question multiple-choice test featured in the Pre-Course Survey. There was no time limit for completing the survey or knowledge testing, though most learners completed within 15 minutes. We recorded all survey and test results for analysis. A key is included for the 10-question multiple-choice test featured in the Pre- and Post-Course Surveys (Appendix 3). A facilitator guide was also created to promote consistency amongst the facilitators and includes a list of additional materials to use for the simulation (Appendix 4).

Statistical analyses were run using SPSS software, version 29.0.1.0 (IBM Corp., Armonk, NY). To analyze overall comfort and knowledge of learners regarding obstetric procedural skills before and after completing the SBML curriculum, as well as the comfort of learners with each individual procedure, we performed Wilcoxon signed-rank testing as these were paired data samples without a normal distribution. If paired data was missing, it was not included in the analysis. Median values were used to minimize the effects of extreme values.

## Results

The curriculum was offered to all first-year residents rotating on L&D at our institution. A total of 11 graduate medical education learners took part in the SBML curriculum, including family medicine, emergency medicine, and OBGYN interns. The SBML curriculum's impact was evaluated by assessing learners' knowledge of obstetric procedural skills, perceived confidence in independent skill performance, and ability to perform each skill using the simulation model. All learners (n=11) were able to independently perform every skill on the simulator, defined as consistent successful performance of membrane rupture, cervical examinations, FSE placement, and IUPC placement. Comfort with each individual skill was assessed using Wilcoxon signed-rank testing and learners’ perceived confidence in performing each skill significantly increased following completion of the SBML curriculum (Table 3). Of note, four learners did not complete the Post-Course Survey due to time constraints on L&D, resulting in n=7. Only the data from those individuals who completed the pre- and post-SBML surveys was included, and this was analyzed using Wilcoxon signed-rank testing in order to pair the pre- and post-SBML survey data for each learner.

**Table 3:** Pre- and post-comfort with individual obstetric procedures Wilcoxon Signed-Rank testing was performed given paired samples of data without a normal distribution. Median values were used to minimize effects of extreme values in the setting of data that is not normally distributed. In this, only paired data was used (n=7). Likert Scale (1 = Strongly Disagree, 2 = Disagree, 3 = Neutral, 4 = Agree, 5 = Strongly Agree).

	Pre-Comfort Median, (IQR) (n=7)	Post-Comfort Median, (IQR) (n=7)	P Value a = 0.05	Test Statistic (Z)	Effect Size (r)
Cervical Exam	3, (2, 3)	4, (4, 5)	0.024*	2.26	0.85
AROM	2 (1, 3)	4, (4, 5)	0.016*	2.41	0.91
IUPC	1 (1, 3)	4, (4, 5)	0.017*	2.38	0.90
FSE	1 (1, 2)	4 (3, 5)	0.026*	2.32	0.88
Cervical Foley	2 (1, 3)	4, (4, 5)	0.017*	2.39	0.90

Learners also completed a written knowledge assessment of obstetric procedural skills. After the SBML curriculum, the median number of questions answered correctly was unchanged from 9/10 (90%) to 9/10 (90%), although the range was smaller in the post-SBML survey. Wilcoxon signed-rank testing revealed no statistically significant difference between these two groups (Table 4).

**Table 4:** Pre- and post-knowledge assessment Wilcoxon Signed-Rank testing was performed given paired samples of data without a normal distribution. Median values were used to minimize effects of extreme values in the setting of data that is not normally distributed. In this, only paired data was used (n=7).

Pre-Knowledge Median, (IQR) (n=7)	Post-Knowledge Median, (IQR) (n=7)	P Value a = 0.05	Test Statistic (Z)	Effect Size (r)
0.9, (0.6, 1.0)	0.9, (0.8, 1.0)	0.102	1.633	0.62

Using the same 5-point Likert Scale as prior, participants additionally expressed their degree of agreement with the statement, “I feel comfortable independently performing obstetric procedural skills.” Following completion of the SBML curriculum, the median ranking increased from 1 (Strongly Disagree) to 4 (Agree). Wilcoxon signed-rank testing revealed a statistically significant difference (Table 5).

**Table 5:** Pre- and post-comfort with independent performance assessment Wilcoxon Signed-Rank testing was performed given paired samples of data without a normal distribution. Median values were used to minimize effects of extreme values in the setting of data that is not normally distributed. In this, only paired data was used (n=7). Likert Scale (1 = Strongly Disagree, 2 = Disagree, 3 = Neutral, 4 = Agree, 5 = Strongly Agree).

Pre-Comfort Median, (IQR) (n=7)	Post-Comfort Median, (IQR) (n=7)	P Value a = 0.05	Test Statistic (Z)	Effect Size (r)
1 (1, 2)	4.0, (4, 5)	0.014*	2.46	0.93

## Discussion

Trainees entering residency programs vary in their knowledge and comfort with obstetric procedural skills and are rarely prepared to perform these skills independently. Our SBML curriculum was well received by learners and improved their comfort performing common obstetric procedural skills as well as their perceived readiness for independent performance, all before they were required to perform a sensitive and invasive exam on a patient. This program, and SBML curricula as a whole, are generalizable, as many other procedural specialties exist where quality and competency are essential for safe and effective patient care. We anticipate the development of future SBML curricula featuring required competency standards within the field of OBGYN, as well as other procedural specialties. 

Although we had trialed the SBML curriculum before implementing it with our learners, we were surprised by how long it took for learners to achieve the predetermined mastery learning objectives set in the course. Because we had only one PROMPT Flex Birthing Simulator, learners could only perform the simulation one at a time. This made it challenging to train large groups of learners, particularly because the curriculum was implemented during learners’ scheduled time on L&D, rather than at times outside of scheduled work hours. These factors resulted in a small sample size, as well as a high dropout rate, with four learners (36%) not completing the post-course assessment due to time constraints secondary to their clinical duties on the L&D floor. As a result, the generalizability of the findings is limited. A larger sample size, especially with the incorporation of multiple study sites, would result in a higher-powered study that is more generalizable to other populations and can detect smaller, albeit still meaningful, differences. In future sessions, we would recommend performing the curriculum during a time with fewer opportunities for interruption, such as during first-year resident orientations, with scheduled times approximately one hour in length for each learner.

Limitations to this curriculum include evaluation data that relies on learners’ subjective assessment of their comfort and ability for independent performance. Additionally, data regarding the number of attempts learners required to achieve mastery (i.e., consistently perform the skill correctly on the simulator) was not collected and may have provided interesting insights into barriers to skill acquisition. Initially, we planned to include a quantitative component in the evaluation data in the form of participants logging the number, type, and success of each procedure they performed on L&D following participation in the course. However, few learners were able to accurately complete this log, and thus the data were not reliable and not included. Future efforts to streamline a process for recording objective procedural data may be useful to assess if and how the SBML curriculum impacts the number and technique of procedures learners performed on L&D. Additionally, we do not yet have data on how this curriculum impacts long-term skill retention; however this could be an area for future study, particularly if the SBML curriculum was implemented in a standard fashion during all incoming first year residents’ orientation, rather than when they each individually began their L&D rotation. In this way, it would be possible to test if learners retained their skills over the elapsed time from completion of the SBML curriculum to performance on L&D months later.

A potential limitation for the implementation of any simulation program is having a model that provides a sufficiently realistic experience for the learner. Realism, or fidelity, of simulation equipment correlates with cost. We chose the Limbs and Things PROMPT Flex Birthing Simulator with Cervical Dilatation and Effacement Module because of the realism, durability, and flexibility of the equipment. Our department previously purchased the base birthing simulator for other obstetric simulation activities, and we purchased the additional module to use for this program using an internal research grant award. There are many other educators who have developed their own models to simulate the procedures we taught in our program. For example, a family medicine team developed a low-cost model using stretch fabric and a doll to simulate various cervical exams and procedures [10]. A benefit of our SBML curriculum and teaching materials is that they can easily be used with less expensive models such as this one.

## Conclusions

Following completion of our SBML curriculum, learners had significantly improved perceived readiness for performing the featured obstetric procedures, as well as improved comfort with independent performance of these skills on L&D. Although the median knowledge score did not change on written knowledge assessments, our sample size may have limited our ability to identify a smaller, but still meaningful, change. Further study is necessary given the small sample size in this cohort, but the results are promising that simulation-based mastery learning may improve how we teach obstetric skills to GME learners. This curriculum provided incoming first-year residents rotating on L&D with a one-on-one training opportunity that improved their confidence and ability to independently perform common obstetric procedures, providing them with practical skills before ever setting foot in a patient room.

## Appendices

Appendix 1: pre-course survey

Please respond to each of the following statements using the below 5-item Likert Scale:

Strongly Disagree = 1 Disagree = 2 Neutral = 3 Agree = 4 Strongly Agree = 5

1. I feel confident in my knowledge of the indications, risks, and contraindications for performing routine obstetric procedures.

2. I feel confident in my ability to perform:      

-Cervical Checks - AROM - IUPC Placement -FSE Placement - Cervical Foley Balloon Placement

3. I feel comfortable independently performing obstetric procedures on Labor & Delivery.

Please answer each of the following questions to the best of your knowledge:

1. When placing an intrauterine pressure catheter, you should place it:

A) Along the interior aspect of the uterus on the same side of the uterus as the placenta

B) Into the vagina so that only the tip of the catheter is past the cervix

C) Along the interior aspect of the uterus on the opposite side of the uterus as the placenta

D) Into the uterus as far as possible until the tip of the catheter re-appears at the cervix

2. When is placing an intrauterine pressure catheter contraindicated?

A) When the fetus is having late decelerations

B) When the patient is in the latent stage of labor

C) When the amniotic sac is intact

D) When meconium is visualized

3. When placing a fetal scalp electrode, you should place it:

A) At the anterior fontanelle

B) On an aspect of the fetal vertex not involving fontanelles or suture lines

C) On the skull suture line

D) At the posterior fontanelle

4. In which circumstance is placing a fetal scalp electrode contraindicated?

A) Maternal hepatitis B infection

B) When meconium-stained amniotic fluid is seen

C) During the active phase of labor

D) Intra-amniotic infection

5. How should the fetal scalp electrode by removed?

A) The cords should be pulled apart gently to release the wire tip

B) The cords should be cut with scissors to release the wire tip

C) A hand should be placed into the uterus to untwist the wire tip manually

D) The cords should be gently pulled out to release the wire tip

6. In what situation would a cervical foley balloon be most helpful in starting an induction of labor?

A) A patient who presents to L&D 5 cm dilated with ruptured membranes

B) A patient who presents to L&D 7 cm dilated with intact membranes

C) A patient who presents to L&D 1 cm dilated with intact membranes

D) A patient who presents to L&D 2 cm dilated with ruptured membranes

7. Which of the following is not a risk of cervical foley balloon placement?

A) Fetal scalp injury

B) Placental disruption

C) Spontaneous rupture of membranes

D) Increased bleeding

8. If after performing AROM (artificial rupture of membranes), you see a fetal heart tracing with deep variable decelerations, you should be most concerned for what labor complication?

A) Placental abruption

B) Uterine rupture

C) Intra-amniotic infection

D) Umbilical cord prolapse

9. Which of the three characteristics of a cervical exam is most important to assess when determining if AROM is appropriate?

A) Cervical dilation

B) Cervical effacement

C) Application of fetal head to cervix

D) Patient’s pain level

10. In which of the following situations is it contraindicated to perform a digital cervical exam?

A) Maternal bacterial vaginosis infection

B) Fetal breech presentation

C) Placenta previa

D) A preterm patient 

Appendix 2: post-course survey

Using the below 5-point Likert Scale, answer each of the following questions:

Strongly Disagree = 1 Disagree = 2 Neutral = 3 Agree = 4 Strongly Agree = 5

1. This simulation improved my understanding of the indications, risks, and contraindications for performing routine obstetric procedures.

2. After participating in this simulation, I feel more confident in my ability to perform:        

Cervical Checks AROM IUPC Placement FSE Placement Cervical Foley Balloon Placement

3. After participating in this simulation, I feel more comfortable with independently performing obstetric procedures on Labor & Delivery.

4. I would recommend this course to my colleagues prior to starting their rotation on Labor & Delivery.

5. This course was useful to me as a provider.

Please answer each of the following questions to the best of your knowledge:

1. When placing an intrauterine pressure catheter, you should place it:

A) Along the interior aspect of the uterus on the same side of the uterus as the placenta

B) Into the vagina so that only the tip of the catheter is past the cervix

C) Along the interior aspect of the uterus on the opposite side of the uterus as the placenta

D) Into the uterus as far as possible until the tip of the catheter re-appears at the cervix

2. When is placing an intrauterine pressure catheter contraindicated?

A) When the fetus is having late decelerations

B) When the patient is in the latent stage of labor

C) When the amniotic sac is intact

D) When meconium is visualized

3. When placing a fetal scalp electrode, you should place it:

A) At the anterior fontanelle

B) On an aspect of the fetal vertex not involving fontanelles or suture lines

C) On the skull suture line

D) At the posterior fontanelle

4. In which circumstance is placing a fetal scalp electrode contraindicated?

A) Maternal hepatitis B infection

B) When meconium-stained amniotic fluid is seen

C) During the active phase of labor

D) Intra-amniotic infection

5. How should the fetal scalp electrode by removed?

A) The cords should be pulled apart gently to release the wire tip

B) The cords should be cut with scissors to release the wire tip

C) A hand should be placed into the uterus to untwist the wire tip manually

D) The cords should be gently pulled out to release the wire tip

6. In what situation would a cervical foley balloon be most helpful in starting an induction of labor?

A) A patient who presents to L&D 5 cm dilated with ruptured membranes

B) A patient who presents to L&D 7 cm dilated with intact membranes

C) A patient who presents to L&D 1 cm dilated with intact membranes

D) A patient who presents to L&D 2 cm dilated with ruptured membranes

7. Which of the following is not a risk of cervical foley balloon placement?

A) Fetal scalp injury

B) Placental disruption

C) Spontaneous rupture of membranes

D) Increased bleeding

8. If after performing AROM (artificial rupture of membranes), you see a fetal heart tracing with deep variable decelerations, you should be most concerned for what labor complication?

A) Placental abruption

B) Uterine rupture

C) Intra-amniotic infection

D) Umbilical cord prolapse

9. Which of the three characteristics of a cervical exam is most important to assess when determining if AROM is appropriate?

A) Cervical dilation

B) Cervical effacement

C) Application of fetal head to cervix

D) Patient’s pain level

10. In which of the following situations is it contraindicated to perform a digital cervical exam?

A) Maternal bacterial vaginosis infection

B) Fetal breech presentation

C) Placenta previa

D) A preterm patient

Appendix 3: multiple choice questions answer key

1: C, along the interior aspect of the uterus on the opposite side of the uterus as the placenta

2: C, when the amniotic sac is intact

3: B, on an aspect of the fetal vertex not involving fontanelles or suture lines

4: A, maternal hepatitis B infection

5: A, the cords should be pulled apart gently to release the wire tip

6: C, a patient who presents to L&D 1 cm dilated with intact membranes

7: A, fetal scalp injury

8: D, umbilical cord prolapses

9: C, application of fetal head to cervix

10: C, placenta previa

Appendix 4: facilitator guide

Course Materials

- Pre-SBML Course Survey

- Computer/AV System to play Standardized Lecture

- Procedural Skills Checklist

- PROMPT Flex Birthing Simulator with the Cervical Dilatation & Effacement Module

   - Stationary Cervical Models (1/40/-2), (4/70/-1)

   - De-flexed Fetal Head Model

   - Flexed Fetal Head Model

   - Dynamic Cervical Model

   - Amniotic Membrane Film

- 30 cc of water with syringe

- Cervical foley Catheter

- Intrauterine Pressure Catheter

- Amniotic Membrane Perforator

- Fetal Scalp Electrode

- Sterile Gloves

- Post-SBML Course Survey

Session Flow

After participants have consented to participation in the study, the Pre-SBML Course Survey is administered. Once this has been completed, the Standardized Lecture is played for the participant. After this has been completed, the simulation-based mastery learning portion of the curriculum takes place. The facilitator should have previously set up the PROMPT Flex Birthing Simulator according to the following:

Examiner Instructions: PROMPT Flex Birthing Simulator Setup

- Use the “deflexed fetal head” for (1/40/-2) cervical exam, cervical foley, 4/70/-1 cervical exam, AROM

- For the 6/100/0 exam, FSE, IUPC placements use the flexed fetal head and the dynamic cervical model.

- For the 10/100/+1 exam, use the dynamic cervical model and flexed fetal head.

- Using the Procedural Skills Checklist, the facilitator should lead the participant through the required skills. Instructions for ensuring the curriculum is administered using a mastery-based learning framework are shown below:

Examiner Instructions: Mastery-Based Learning Framework

- Before advancing to the next skill, the learner must complete the required competencies from the ongoing skill. 

- The facilitator should be present and provide real-time feedback while the learner is engaged in the SBML curriculum. 

- The facilitator can provide guidance but the learner must be able to independently complete each skill to complete the SBML curriculum. 

- If a learner incorrectly answers a question or performs a skill incorrectly, the facilitator should correct them and prompt them to re-attempt the skill, as well as ask the question again until the learner demonstrates the ability to both perform the skill and understand the associated concept.

After learners complete the required skills, the Post-SBML Course Survey is administered. Once this has been completed, participants are thanked for their participation in the study and can be excused.

## References

[1] (2024). First and second stage labor management. ACOG Clinical Practice Guideline No. 8. Obstet Gynecol.

[2] de Klerk HW, Boere E, van Lunsen RH, Bakker JJ (2018). Women's experiences with vaginal examinations during labor in the Netherlands. J Psychosom Obstet Gynaecol.

[3] Ennen CS, Satin AJ (2010). Training and assessment in obstetrics: the role of simulation. Best Pract Res Clin Obstet Gynaecol.

[4] Gomez Slagle HB, Hoffman MK, Fonge YN, Caplan R, Sciscione AC (2022). Incremental risk of clinical chorioamnionitis associated with cervical examination. Am J Obstet Gynecol MFM.

[5] McGaghie WC, Issenberg SB, Cohen ER, Barsuk JH, Wayne DB (2011). Medical education featuring mastery learning with deliberate practice can lead to better health for individuals and populations. Acad Med.

[6] Cassimatis IR, Peaceman AM, Gerber S (2023). Simulation-based mastery learning improves physical exam-indicated cerclage skills among OB/GYN residents. Am J Obs Gyn.

[7] Blumenfeld A, Velic A, Bingman EK, Long KL, Aughenbaugh W, Jung SA, Liepert AE (2020). A mastery learning module on sterile technique to prepare graduating medical students for internship. MedEdPORTAL.

[8] Satin AJ (2018). Simulation in obstetrics. Obstet Gynecol.

[9] Whittington JR, Shnaekel KL, Ramseyer AM, Cato M, Ounpraseuth S, Hughes DS, Magann EF (2022). Longitudinal assessment of obstetrics and gynecology resident perceptions and comfort following cerclage placement simulation. J Matern Fetal Neonatal Med.

[10] Little SH, Heinrich LE, Sen A, Gold KJ (2023). Learning obstetrical cervical exam skills: development of a novel model to demystify blind procedures. Fam Med.

[11] Parameshwar PS, Bianco K, Sherwin EB (2022). Mixed methods evaluation of simulation-based training for postpartum hemorrhage management in Guatemala. BMC Pregnancy Childbirth.

[12] Gavin NR, Satin AJ (2017). Simulation training in obstetrics. Clin Obstet Gynecol.

[13] Guskey TR (2007). Closing achievement gaps: revisiting Benjamin S. Bloom's "learning for mastery". J Adv Acad.

[14] McGaghie WC, Issenberg SB, Cohen ER, Barsuk JH, Wayne DB (2011). Does simulation-based medical education with deliberate practice yield better results than traditional clinical education? A meta-analytic comparative review of the evidence. Acad Med.

